# Disutility of injectable therapies in obesity and type 2 diabetes mellitus: general population preferences in the UK, Canada, and China

**DOI:** 10.1007/s10198-022-01470-w

**Published:** 2022-05-08

**Authors:** Phil McEwan, James Baker-Knight, Björg Ásbjörnsdóttir, Yunni Yi, Aimee Fox, Robin Wyn

**Affiliations:** 1 Health Economics and Outcomes Research Ltd, Cardiff, Wales, UK; 2 Novo Nordisk A/S, Søborg, Denmark; 3 Adelphi Values PROVE, Cheshire, England, UK

**Keywords:** Time trade-off (TTO), Type 2 diabetes mellitus (T2DM), Obesity, Insulin, GLP-1 receptor agonist (GLP-1 RA), I10

## Abstract

**Introduction:**

Once-daily and once-weekly injectable glucagon-like peptide-1 receptor agonist therapies (GLP-1 RAs) are established in obesity and type 2 diabetes mellitus (T2DM). In T2DM, both once-daily and once-weekly insulin are expected to be available. This study elicited utilities associated with these treatment regimens from members of the general public in the UK, Canada, and China, to quantify administration-related disutility of more-frequent injectable treatment, and allow economic modelling.

**Methods:**

Two anchor states (no pharmacological treatment), and seven treatment states (daily oral tablet and generic injectable regimens of variable frequency), with identical outcomes were tested A broadly representative sample of the general public in each country participated (excluding individuals with diabetes or pharmacologically treated obesity). An adapted *Measurement and Valuation of Health* protocol was administered 1:1 in web-enabled interviews by trained moderators: visual analogue scale (VAS) as a “warm-up”, and time trade-off (TTO) using a 20-year time horizon for utility elicitation.

**Results:**

A total of 310 individuals participated. The average disutility of once-daily versus once-weekly GLP-1 RA was − 0.048 in obesity and − 0.033 in T2DM; the corresponding average disutility for insulin was − 0.064. Disutilities were substantially greater in China, relative to UK and Canada.

**Discussion:**

Within obesity and T2DM, more-frequent treatment health states had lower utility. Scores by VAS also followed a logical order. The generated utility values are suitable for use in modelling injectable therapy regimens in obesity and T2DM, due to the use of generic descriptions and assumption of equal efficacy. Future research could examine the reasons for greater administration-related disutility in China.

**Supplementary Information:**

The online version contains supplementary material available at 10.1007/s10198-022-01470-w.

## Introduction

Injectable glucagon-like peptide-1 receptor agonist therapies (GLP-1 RAs) are an effective and established modern treatment option for patients with obesity or type 2 diabetes mellitus (T2DM) [1, 2]. Therapies of the GLP-1 RA class are typically administered as once-daily or once-weekly injectables, and are associated with outcomes such as improved blood glucose control, reduced body weight, and cardiovascular benefits [3, 4]. Within the treatment pathway for T2DM, GLP-1 RAs are typically positioned between once-daily oral metformin and once- or multiple-daily injectable insulin [5].

However, regular injectable therapy may impact health-related quality of life (HRQoL), and may be associated with reduced adherence to treatment [6–9]. From an economic evaluation perspective, it is important to understand and quantify the potential impact that this may have on patients, particularly as treatment regimens can include either once-weekly or once-daily injections. Therefore, data on the QoL impact of therapeutic regimens with different frequencies of injections are increasingly required for cost–utility analysis of new therapy regimens.

Quantifying patient HRQoL for economic evaluation purposes often relies on health state utility values, which represent individuals’ preferences for different health states. A frequently used method of utility data collection is through the use of generic, preference-based measures such as the EuroQol five-dimensions (EQ-5D) instrument. Within the EQ-5D, a health state description (i.e., a questionnaire filled by a patient) is paired with a health state valuation (i.e., a utility value from a value set previously generated by members of the general population) [10]. This indirect method is specified by the reference case of the National Institute of Health and Care Excellence (NICE) in the UK, and many other health technology assessment (HTA) bodies, including those in Canada and China [11–13].

However, using indirect methods may not be indicated in situations where the available measures do not adequately capture differences in utility. For example, differences in convenience between injectable treatment regimens may result in reductions in utility (i.e., disutilities) that are not captured within the five domains of the EQ-5D questionnaire (mobility, self-care, usual activity, pain/discomfort, and anxiety/depression) [14]. In such cases, a direct method of deriving accurate utility values can be explored, such as a mathematical exercise like the standard gamble (SG) or time trade-off (TTO) [15].

Preferences for differing injectable regimens in T2DM have previously been explored using direct utility elicitation methods with patients in the UK and Italy [16–18]. However, HTA bodies typically prefer for the valuation of health states to be carried out with members of the general population in their country, to reflect societal preferences [11–13]. Therefore, there is an unmet need for data on preferences related to the administration of injectable therapy regimens in obesity and T2DM that have been elicited by members of the general public, and that can be used to evaluate the cost-utility of therapies in these two major chronic health conditions.

The primary objective of this study was to elicit utilities associated with once-weekly and once-daily injectable therapies for obesity and T2DM from members of the general public in three countries, and therefore quantify any administration-related disutility of more-frequent injectable therapy. Countries of interest were the UK, Canada, and China: three countries experiencing increasing prevalence of these metabolic disorders, and where publicly derived utility values are key for economic modelling of new therapies [19–21].

## Methods

### Overview of study design

To align with the HTA requirements described above, a TTO utility elicitation study was designed to estimate the preferences for variably administered injectable therapies for patients with obesity and T2DM from members of the general population in three countries (UK, Canada, and China) [22, 23]. Each TTO exercise followed the standard *Measurement and Valuation of Health* (*MVH*) protocol used by EuroQol for the valuation of their three-level questionnaire (EQ-5D-3L) in the UK, with minor adaptations (see below) [11, 23–25].

### Development of health state descriptions

Each health state description was developed according to characteristics of relevant therapy regimens of interest for this study. Health state descriptions were developed in plain language, to ensure that they could easily be understood by a member of the general public without a clinical background [26].

Medical experts and health economics and outcomes research professionals in each country were consulted during the development stage. This ensured that the depicted treatment regimens and outcomes were relevant to each market. When appropriate, each health state was translated, and the accuracy and comprehensibility of each translation were then confirmed by fluent speakers.

### Health state descriptions for valuation

A total of nine health state descriptions were developed and valued (see Table 1 and Supplementary material), based on treatment regimens that are currently used in practice, or anticipated to soon become available (that is, once-weekly insulin).

**Table 1:** Summary of health state descriptions

Health state	Diet and exercise	Oral therapy	Injectable therapy	Blood glucose testing	Disease outcome
Obesity anchor	As appropriate for obesity	✕	✕	✕	BMI controlled (borderline of obesity)
GLP-1 RA QW		Once-daily	Once-weekly	✕	
GLP-1 RA QD			Once-daily	✕	
T2DM anchor	As appropriate for T2DM	✕	✕	✕	Blood glucose controlled (borderline of diabetes)
GLP-1 RA QW		Once-daily	Once-weekly	✕	
GLP-1 RA QD			Once-daily	✕	
Insulin QW			Once-weekly	Once-weekly	
Insulin QD			Once-daily	Once-daily	
Insulin BB			Four times daily	Four times daily	

*BB* basal–bolus administration, *GLP-1 RA* glucagon-like peptide-1 receptor agonist, *QD* once-daily administration, *QW* once-weekly administration

✕: therapy or monitoring requirement was not a part of the specified treatment regimen

Two anchor health state descriptions were developed for obesity and T2DM. The purpose of the anchor health state descriptions was to determine the baseline utility of living with the controlled disease in each condition. These descriptions depicted obesity or T2DM being controlled with an appropriate diet and exercise only.

The other health state descriptions began with the same information on obesity or T2DM as the anchor descriptions and then specified the use of oral and injectable therapies. Herein, these health states are referred to as treatment health states. Two treatment health state descriptions depicted obesity and five depicted T2DM.

Between each treatment description, injectable therapy regimens varied in terms of frequency of administration (and requirement for blood glucose testing), while oral therapy regimens were identical. The depicted oral therapies represented orlistat or metformin once-daily (for obesity and T2DM, respectively), while the depicted injectable therapies were generic representations of GLP-1 RA and insulin regimens. Insulin and GLP-1 RA therapies in T2DM were included within separate health states, due to the difference in monitoring requirements associated with insulin therapy. Specific characteristics of injection devices and handling processes were not specified, to ensure generalisability of results.

Disease outcomes were identical between each health state within the conditions. Within the obesity health states, the disease outcome was described as a controlled BMI (at the threshold of obesity). For T2DM, the disease outcome was described as controlled blood glucose (at a level between pre-diabetes and diabetes).

### Participants in utility elicitation interviews

Members of the general public were initially recruited by phone or email, before participation in 1:1 interviews. Care was taken to recruit participant samples that were broadly representative of the general public in terms of factors such as gender, age, employment, and education, as well as geographical location within the three countries. A minimum sample size of 100 was sought in each country.

Participants were required to fulfil the following inclusion criteria: (1) be over the age of 18 years; (2) no diagnosis of diabetes (of any type); (3) not receiving a prescribed pharmacological treatment for obesity; (4) no participation in market research within the last 6 months; and (5) no conflict of interest (defined as employment in healthcare or market research).

Individuals with diabetes or pharmacologically treated obesity were excluded, as having prior experience of the health states in question has been known to affect utility valuation [10, 22]. In addition, valuation by the general population rather than affected individuals is considered standard in many regions with publicly funded healthcare systems [10, 11, 22].

Prior to the initiation of the main study, a pilot study was conducted in the UK to ensure the comprehensibility of health state descriptions and interview methods. Pilot study interviews were conducted with 10 participants. No participant reported difficulty in understanding the health states or the TTO procedure itself. Some minor revisions were suggested for wording and formatting, which were subsequently included in the final health states.

### Utility data collection

At the time of the TTO fieldwork, COVID-19 restrictions were in place in all three countries. Therefore, participants were each interviewed using 1:1 web-enabled interviews. Interviews were conducted by trained TTO moderators, using pre-programmed survey links (including visual aids). Survey links were consistent between countries, with the exception of translation, as required. This guaranteed consistency of presentation and data collection.

The interviews took place between 2nd February and 26th March 2021. All participants provided written informed consent prior to interview.

### Utility elicitation process

In accordance with the MVH protocol used by EuroQol [23], visual analogue scale (VAS) exercises were first conducted to ensure that participants understood the concept of valuing health states. During the VAS exercise, participants were asked to rate each health state on the VAS scale between 0 (death) and 100 (full health). These exercises also allowed for validation of TTO utility responses after fieldwork.

Following the VAS exercises, TTO exercises were conducted for each health state. Each TTO exercise comprised questions prompting the participants to select between *x* years in full health, and 20 years in the health state (rather than 10 years within the *MVH* protocol, as per published outcomes research in T2DM) [18, 27]. At each step, if a participant selected full health, *x* was decreased, and if a participant selected the health state, *x* was increased. Participants were able to report reaching a point of indecision at any step. The smallest possible increment was 1 month (on a scale of 20 years; therefore, a difference in utility of ± 0.00417).

The minimum and maximum possible values were *x* = 0 years (utility 0.0), and *x* = 20 years (utility 1.0). Values below 0.0 (i.e., valuation of health states as being worse than death) have been observed to be unrealistic and rare in this therapy area [18], and therefore, the interview structure was designed in order that these were not possible (unlike in the *MVH* protocol, which was designed for valuation of a much wider range of health states) [18].

To avoid systematic bias, the health states were presented in forward (e.g., most-to-least frequent administration) or reverse order (least-to-most frequent administration), randomly to each participant; each possible presentation order was assigned to a respondent identification number prior to recruitment. The order of presentation of obesity and T2DM health states was also randomised, in accordance with the above procedure (see Table 2). However, to minimise participant fatigue, for each participant, the presentation order was kept consistent between the VAS and TTO exercises.

**Table 2:** The four health state orders to which participants were assigned for VAS and TTO exercises

#1	T2DM intro + anchor	GLP-1 QW	GLP-1 QD	Insulin QW	Insulin QD	Insulin BB	Obesity intro + anchor	GLP-1 QW	GLP-1 QD
#2	T2DM intro + anchor	Insulin BB	Insulin QD	Insulin QW	GLP-1 QD	GLP-1 QW	Obesity intro + anchor	GLP-1 QD	GLP-1 QW
#3	Obesity intro + anchor	GLP-1 QW	GLP-1 QD	T2DM intro + anchor	GLP-1 QW	GLP-1 QD	Insulin QW	Insulin QD	insulin BB
#4	Obesity intro + anchor	GLP-1 QD	GLP-1 QW	T2DM intro + anchor	Insulin BB	Insulin QD	Insulin QW	GLP-1 QD	GLP-1 QW

*BB* basal–bolus administration, *GLP-1 RA* glucagon-like peptide-1 receptor agonist, *QD* once-daily administration, *QW* once-weekly administration, *TTO* time trade-off, *VAS* visual analogue scale

Interview moderators were briefed to revisit illogical responses (e.g., an instance where a health state with an increased burden of injections was rated higher than a state with identical health outcomes but lower burden). In these cases, interview participants were able to freely amend their valuations.

### Data analysis

Descriptive analyses were conducted on the TTO results, including calculation of means, standard deviations, medians, and interquartile ranges for each health state. Utility values were calculated by dividing *x* by 20 (e.g., a result of 10 years and 6 months divided by 20 years gives a utility value of 0.525). Disutility values were calculated by simply subtracting the mean utility value in question from a reference utility value.

Subgroup analyses were conducted according to major demographic criteria (gender and age) to determine if utility values are substantially different in demographic subgroups. In addition, the effect of extreme responses (0.0 or 1.0) was examined by removing such values and recalculating utility values, as a sensitivity analysis. Two further sensitivity analyses were also conducted by removing the highest 5% and lowest 5% of values within each health state, and by removing values that were at least two standard deviations above or below each corresponding health state mean.

In addition, a simple logic check was conducted, where each health state utility value was compared to one other, according to a logical ordering that was assumed prior to the conduct of research. Specifically, these assumptions were: utility of anchor state is not lower than utility of once-weekly injection; utility of once-weekly injection is not lower than utility of once-daily injection; utility of once-daily injection is not lower than utility of basal–bolus injection (where relevant). Following this logic check, a further sensitivity analysis was conducted by removing any illogical responses identified (see Supplementary material).

Utility values and disutility estimates presented here are derived from TTO only, as this is the method typically preferred by HTA bodies [23]. Scores from VAS are also presented for comparison purposes.

## Results

### Characteristics of TTO interview participants

A total of 310 participants completed the TTO interviews: 110 in the UK, 100 in Canada, and 100 in China. Demographic characteristics of these participants are presented in Table 3.

**Table 3:** Demographic characteristics of TTO interview participants in the UK, Canada, and China

Characteristic	UK *n* = 110	Canada *n* = 100	China *n* = 100
	*n* (%)	*n* (%)	*n* (%)
Male	51 (46.4%)	49 (49.0%)	50 (50.0%)
Female	59 (53.6%)	51 (51.0%)	50 (50.0%)
18 to 20 years	3 (2.7%)	5 (5.0%)	6 (6.0%)
21 to 29 years	21 (19.1%)	16 (16.0%)	25 (25.0%)
30 to 39 years	24 (21.8%)	15 (15.0%)	38 (38.0%)
40 to 49 years	19 (17.3%)	22 (22.0%)	17 (17.0%)
50 to 59 years	23 (20.9%)	18 (18.0%)	11 (11.0%)
≥ 60 years	20 (18.2%)	24 (24.0%)	3 (3.0%)
Married/cohabiting	49 (44.5%)	52 (52.0%)	58 (58.0%)
Previously married/separated	21 (19.1%)	10 (10.0%)	8 (8.0%)
Unmarried	40 (36.4%)	38 (38.0%)	34 (34.0%)
Working	83 (75.5%)	65 (65.0%)	69 (69.0%)
Not working	17 (15.5%)	18 (18.0%)	24 (24.0%)
Retired	10 (9.1%)	38 (38.0%)	7 (7.0%)
School education	44 (40.0%)	18 (18.0%)	27 (27.0%)
Trade/technical/vocational training	31 (28.2%)	23 (23.0%)	33 (33.0%)
Degree-level education	35 (31.8%)	59 (59.0%)	40 (40.0%)
North of England	27 (24.5%)		
Midlands of England	10 (9.1%)		
South of England (including London)	33 (30%)		
Scotland	30 (27.3%)		
Wales	10 (9.1%)		
Ontario		34 (34.0%)	
Quebec		36 (36.0%)	
British Columbia		12 (12.0%)	
Alberta		8 (8.0%)	
Other*		10 (10.0%)	
Northern China			13 (13.0%)
Eastern China			38 (38.0%)
Southern China			34 (34.0%)
Central China			9 (9.0%)
Western China			6 (6.0%)

Northern China includes Beijing, Datong, Jilin, Harbin, Shenyang, Tsitsihar, and Zaozhuang. Eastern China includes Changshu, Ganzhou, Kunshan, Luan, Nanjing, Ningbo, Shanghai, Shangyao, Shuqian, Wenzhou, Wuhu, Wuxi, Xiamen, Xuzhou, and Yantai. Southern China includes Beihai, Changsha, Dongguan, Foshan, Guangzhou, Hengyang, Jieyang, Liuzhou, Loudi, Nanning, Qionghai, Shantou, Shaoyang, Zhanjiang, and Zhaoqing. Central China includes Hengyang, Jiaozuo, Jiujiang, Linzhou, Sanming, Yancheng, and Zhumadian. Western China includes Bazhong, Chengdu, Kunming, Yuxi, and Zhaotong

*Other Canadian provinces include Saskatchewan, Nova Scotia, Manitoba, and New Brunswick

### Utility values for anchor and treatment health states in obesity and T2DM

**Fig. 1:**
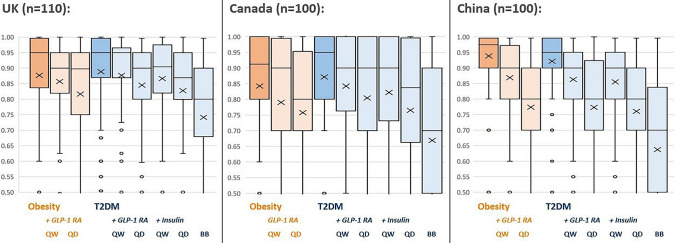
TTO utility values* in obesity and T2DM, in the UK, Canada, and China. *Values between 0.0 and 1.0 were collected; 0.5 to 1.0 are presented here to aid the interpretation of differences between means. Please see supplementary material for full chart presenting values 0.0 to 1.0. *BB* basal–bolus, *GLP-1 RA* glucagon-like peptide-1 receptor agonist, *QD* daily, *QW* weekly, *TTO* time trade-off.  × mean, — median value, ◻ interquartile range (first quartile to third quartile), ○ outlier value (outside 1.5 × interquartile range)

Key calculated disutility values from participants in each country are presented in Table 4. Within obesity and within T2DM, utility values logically followed the expected ordering. Health states containing pharmacological treatment, especially more-frequent treatment, were valued lower by the participants. Also, as expected, the disutility of more-frequent insulin treatment (where blood glucose testing was also required) was greater than the disutility of more-frequent GLP-1 RA treatment in T2DM. Detailed utility results are presented in Fig. 1 and Table 5, and Supplementary material.

**Table 4:** Differences between mean utility values caused with the use of more-frequent therapy regimens in the UK, Canada, and China

Condition	Less frequent therapy	More frequent therapy	Utility difference
UK (*n* = 110)			
Obesity	Once-weekly GLP-1 RA	Once-daily GLP-1 RA	− 0.0404
T2DM	Once-weekly GLP-1 RA	Once-daily GLP-1 RA	− 0.0316
	Once-weekly insulin	Once-daily insulin	− 0.0389
	Once-daily insulin	Multiple-daily insulin	− 0.0861
Canada (*n* = 100)			
Obesity	Once-weekly GLP-1 RA	Once-daily GLP-1 RA	− 0.0329
T2DM	Once-weekly GLP-1 RA	Once-daily GLP-1 RA	− 0.0376
	Once-weekly insulin	Once-daily insulin	− 0.0569
	Once-daily insulin	Multiple-daily insulin	− 0.0958
China (*n* = 100)			
Obesity	Once-weekly GLP-1 RA	Once-daily GLP-1 RA	− 0.0950
T2DM	Once-weekly GLP-1 RA	Once-daily GLP-1 RA	− 0.0900
	Once-weekly insulin	Once-daily insulin	− 0.0947
	Once-daily insulin	Multiple-daily insulin	− 0.1230

*GLP-1 RA* glucagon-like peptide-1 receptor agonist

**Table 5:** TTO utility values* in obesity and T2DM, in the UK, Canada, and China

	Obesity			T2DM					
	Anchor	GLP-1 RA		Anchor	GLP-1 RA		Insulin		
		QW	QD		QW	QD	QW	QD	BB
*UK (n = 110)*									
Mean	0.877	0.857	0.816	0.888	0.877	0.845	0.866	0.827	0.741
SD	0.165	0.176	0.194	0.161	0.177	0.180	0.179	0.183	0.210
*Canada (n = 100)*									
Mean	0.843	0.790	0.757	0.872	0.842	0.805	0.822	0.765	0.669
SD	0.222	0.253	0.268	0.200	0.216	0.233	0.229	0.263	0.297
*China (n = 100)*									
Mean	0.939	0.869	0.774	0.921	0.863	0.773	0.855	0.760	0.637
SD	0.091	0.146	0.198	0.103	0.146	0.200	0.153	0.204	0.249

*BB* basal–bolus, *GLP-1 RA* glucagon-like peptide-1 receptor agonist, *QD* daily, *QW* weekly, *SD* standard deviation, *TTO* time trade-off

*Corresponding median and IQR values presented in Supplementary material

### Subgroup analyses, sensitivity analysis, and logic check of utility values in obesity and T2DM

Utility values elicited by TTO from subgroups of participants are presented in Supplementary material. Values in the tested subgroups were broadly consistent with those from the full participant samples in each country.

Utility values estimated in sensitivity analyses are presented in Supplementary material. Mean values were broadly consistent between the full analysis and the three sensitivity analyses, and continued to be logically ordered.

Considering each participant’s individual responses, a logic check was also applied, where each health state utility value was compared to one other, according to a logical ordering that was assumed prior to the conduct of research (see Supplementary material). Specifically, these assumptions were: utility of anchor state is not lower than utility of once-weekly injection; utility of once-weekly injection is not lower than utility of once-daily injection; utility of once-daily injection is not lower than utility of basal–bolus injection (where relevant). Comparing each individual response to these assumptions, the number of illogical responses (where a less-convenient health state had higher utility) was 89 from a total of 2170 comparisons (4.1%). A further sensitivity analysis where these 89 responses were removed was again broadly consistent with the full analysis and continued to be logically ordered; therefore, all responses continued to be included in the full analysis.

### VAS scores for anchor and treatment health states in obesity and T2DM

Scores given by VAS are presented in Supplementary material. Scores by VAS logically followed the expected ordering, similarly to the TTO utility values, in each country.

## Discussion

There is a marked need for research into the QoL impact of variably administered injectable therapies in metabolic diseases such as obesity and T2DM. To the authors’ knowledge, this is the first study to elicit utility values for once-weekly and once-daily therapies (without specifying injection device) in obesity or T2DM, in the UK, Canada, and China: three countries experiencing increasing prevalence of these metabolic disorders, and therefore increasing need for effective and convenient treatment regimens [19–21].

Utility values were elicited using TTO from members of the general public in three countries, and all values followed the logical and expected ordering, confirming that the use of injectable therapy, and especially more-frequent injectable therapy, led to an associated disutility due to administration factors. This research, therefore, offers utility value estimates that are suitable for use in economic modelling when assessing any therapy for T2DM or obesity where there is variation in the frequency of injectable use. This is due to the generic nature of the health state descriptions that were valued, and the explicit assumption of equal efficacy across health states.

Utility values collected for borderline obesity without pharmacological treatment in the current study (0.84–0.94) appear broadly consistent with and are reinforced by those from a review of previous literature, where the majority of utility values for obesity (elicited from patients and the general public by a variety of methods, and not necessarily defined as controlled obesity) were between 0.70 and 0.85 [28].

Preferences for differing injectable regimens in T2DM have also previously been explored among patients in the UK and Italy using direct utility elicitation [16–18]. Administration-related disutility values collected from patients in these studies are generally smaller than those collected in the current study (see Table 6 in Supplementary material). The disutility of weekly injectable GLP-1 RA treatment added to oral treatment has previously been estimated as − 0.013 to − 0.020 in Italy [16], and − 0.010 to − 0.030 in the UK (dependent on injection device) [18], versus − 0.011, − 0.030, or − 0.058 in the current study (dependent on country). The disutility of daily versus weekly injectable treatment in T2DM has been estimated as − 0.023 in Scotland [17], versus − 0.032, − 0.037, or − 0.090 (GLP-1 RAs), or − 0.039, − 0.057, or − 0.095 (insulins) in the current study. The use of patient preferences (from individuals living with T2DM) rather than general public preferences may have contributed to a reduction in perceived disutility in these studies, due to the adaptation of these individuals to treatment regimens that they have received [22]. To the authors’ knowledge, no comparable published literature on the disutility of injectable treatment exists in the area of obesity.

In the current study, disutility values with more-frequent treatment in the UK and Canada appeared to be consistent. However, in China, the corresponding disutility of more-frequent treatment was substantially greater, suggesting that the perceived QoL impact of these therapy regimens is greater in a Chinese population. This discrepancy in the magnitude of disutility between China and the UK and Canada in the current study (and the previously published value from Italy) may be explained by differences in lifestyle or culture. However, the available literature on health preferences in Chinese versus Western populations does not conclusively suggest the direction or magnitude of difference that should be expected between these populations, in an exercise such as TTO [29–31]. Further research into this topic should be conducted.

Scores gathered by VAS in the current study for comparison confirmed the logical ordering of values gathered by TTO, and also showed a greater difference between most- and least-preferred health states in China, relative to in the UK and Canada. The presence of this discrepancy in both TTO and VAS results suggests that this is not the result of a systemic bias (for example, a difference in how TTO or VAS exercises were administered in this country).

Therefore, these administration-related disutility estimates offer health economists important inputs for any future cost–utility analysis in this area. In particular, these estimates may be beneficial for analyses in the UK, Canada, and China, in which data from the general population of each country itself may be preferred for healthcare decision-making.

The strengths of this study include having elicited utility values using an established TTO protocol (with VAS warm-up) which is an accepted approach by HTA bodies in the UK, Canada, and China when generic preference-based instruments are not appropriate [11–13, 23]. Utility elicitation interviews were carried out with members of the general public in each of three countries (representing Europe, North America, and Asia), as preferred by relevant HTA bodies [11–13]. This sample size of ≥ 100 participants is also broadly consistent with prior research in T2DM on utility elicitation [16–18, 32–34], or patient preferences in general [35, 36]. In addition, a moderated 1:1 interview method (with highly consistent electronic data collection) was used, which represents the gold standard for health state valuation [23]. A set of pilot interviews conducted in the UK as part of this study provided additional prior validation of this methodology. Additionally, the use of generic descriptions of treatment regimens allows the estimated disutility values to be generalised across therapy compounds and injection devices.

The limitations of this study are inherent to the TTO utility valuation methodology. Members of the general public (and especially those without diabetes or medically treated obesity) reading written health state descriptions are likely not able to fully visualise and understand the experience of living with a given health condition [37]. In addition, these participants may not readily understand the adaptation that affected individuals may make to their condition over time [10, 37]. The presence of the COVID-19 pandemic and the virtual nature of the interviews during TTO valuation may also have affected preference results in a way that is difficult to account for.

Lengthy health state descriptions were required to convey necessary medical and pharmacological information to general public participants, which could have proved a barrier to full understanding. However, these descriptions were designed with a clear and repetitive structure (with key differences between treatment regimens being highlighted) to reduce respondent fatigue, and comprehensibility was additionally confirmed in pilot interviews with members of the general public.

While the UK sample appeared broadly representative of the wider UK population in key demographic characteristics [38–42], the Canada sample was more often degree-educated than the wider Canadian population (59% versus ~ 32%) [43], and the China sample was younger (3% aged ≥ 60 years versus ~ 19%) and more often degree-educated than the corresponding national population (40% vs ≤ 20%) [44, 45]. These characteristics may influence participants’ perceptions and preferences, and therefore the utility values that are elicited.

## Conclusion

From this TTO utility elicitation study, there are logical and consistent results across the general population of the UK, Canada, and China that show greater disutility associated with more frequently administered injectable therapies. In UK and Canada, the disutilities appeared similar; in China, disutilities were greater.

Accounting for more-frequent injections, when assessing the cost–utility of regimens for patients with obesity or T2DM, is therefore likely to be an important consideration.

This research, therefore, offers new estimates that may contribute to a more accurate understanding of the burden of frequent injectable therapy in these countries, where treatment needs are expected to increase. Our utility values are suitable for use in modelling across the injectable GLP-1 RA and insulin classes in these three countries, due to the generic nature of the health state descriptions that were valued, and how utility values were derived from members of the general public in each region.

Future research could examine the reasons behind the greater administration-related disutility seen with injectable therapy in China, relative to Western countries, and the implications that this difference could have for economic modelling, and the development of injectable therapies in this country.

## Supplementary Information

Below is the link to the electronic supplementary material.

Supplementary file1 (DOCX 560 KB)
