# Supplementary material for: Disutility of injectable therapies in obesity and type 2 diabetes mellitus: general population preferences in the UK, Canada, and China
Source: Eur J Health Econ. 2022 May 8;24(2):187–96. doi: 10.1007/s10198-022-01470-w (PMC9080344; doi:10.1007/s10198-022-01470-w)
Supplement: Supplementary file 1 — Supplementary file1 (DOCX 560 KB) [file 10198_2022_1470_MOESM1_ESM.docx]

# Supplementary material

## Comparison of TTO disutility values with prior research

Table 6. Differences between mean utility values caused with use of more frequent therapy regimens, as reported in the current study and in previous literature[16-18]

| Health state comparison | | Obesity | T2DM | | | |
| --- | --- | --- | --- | --- | --- | --- |
|  |  | Current  study | Current  study | Boye  *et al*.  2019 | Boye  *et al*.  2011 | Matza  *et al*.  2017 |
| Less frequent  health state | **More frequent health state** | *Valuation by*  *general public* | | *Valuation by*  *T2DM patients* | | |
|  |  | *UK, Canada, China^*^* | | *Italy* | *Scotland* | *UK* |
| Oral  treatment | Once-weekly  GLP-1 RA | −0.020  −0.053  −0.070 | −0.011  −0.030  −0.058 | −0.013†  −0.020† | n/a | −0.010^†^  −0.030^†^ |
| Once-weekly  GLP-1 RA | Once-daily  GLP-1 RA | −0.041  −0.033  −0.095 | −0.032  −0.037  −0.090 | n/a | −0.023^‡^ | n/a |
| Oral  treatment | Once-weekly  insulin | n/a | −0.022  −0.050  −0.066 | n/a | n/a | n/a |
| Once-weekly  insulin | Once-daily  insulin | n/a | −0.039  −0.057 −0.095 | n/a | −0.023^‡^ | n/a |

*Values from the current study are presented in the following order within each comparison: UK, Canada, China.

†Heath states in Boye *et al*. 2019 and Matza *et al*. 2017 included more than one GLP-1 RA injection device; therefore, multiple values are presented.

‡Health states in Boye *et al*. 2011 were not specific to GLP-1 RA or insulin treatment; therefore, this value is presented in both the GLP-1 RA and insulin sections.

“n/a” indicates that the health state comparison was not conducted or not reported.

## Obesity introductory text and health state descriptions

**Introductory text**

Obesity is a condition where the body stores too much fat tissue, meaning that body weight and waist size increase to a degree that affects a person’s health and their ability to complete activities in their daily life. For example, a person who is 176 cm tall and who weighs 89 kg or more would be considered obese. Being obese increases the risk of complications that affect the heart (such as high blood pressure or cardiovascular disease), joints (such as osteoarthritis), risk of stroke, cancer and diabetes. In addition, a person with obesity may experience difficulties completing activities such as work, due to their condition. Therefore, reducing weight from an obese level is positive for health.

**Anchor (diet and exercise only)**

| - You have obesity. - Because of your obesity, your doctor has advised you to monitor your diet and exercise regularly. - You follow your doctor’s recommendations about your diet. - You count the number of calories in the foods that you eat, and you make sure that you do not exceed the daily limit that your doctor has recommended. - For certain foods, you limit the amount you eat, or how often you eat them. - You follow your doctor’s recommendations about exercise. - You do aerobic exercise (such as brisk walking) for 2½ hours per week, spread across three or more days. - You consistently follow this guidance, and your weight is maintained at a level between being obese and overweight, without further increasing or decreasing. - Despite following the guidance, at this level, your risk of obesity-related complications is not significantly increased compared to a person who is overweight, but not obese, and you are more easily able to complete activities such as work. |
| --- |

**#1 GLP-1 RA QW (once-weekly + oral therapy)**

| - You have obesity. - Because of your obesity, your doctor has advised you to monitor your diet and exercise regularly. - You follow your doctor’s recommendations about your diet. - You count the number of calories in the foods that you eat, and you make sure that you do not exceed the daily limit that your doctor has recommended. - For certain foods, you limit the amount you eat, or how often you eat them. - You follow your doctor’s recommendations about exercise. - You do aerobic exercise (such as brisk walking) for 2½ hours per week, spread across three or more days. - Your doctor has also advised you to take medication that reduces your weight. - You take tablets and injections. - You take daily tablets prescribed by your doctor. - You give yourself an injection **once a week**, from a pre-filled injection device. (One injection per week.) - You may choose the time of day when you give yourself the injection. - Before beginning to use the injection device, you may store it in your refrigerator or at room temperature. - Before giving yourself the injection, you must place a new needle on the device and remove both protective caps from the needle. Because of the protective cap, you do not directly handle the needle at any point. You must then dial up to priming dose, and press the button to test whether the medicine can clearly pass through this needle. When this is confirmed, you can dial up to the dose that your doctor has advised, and then administer the injection. - After you give yourself the injection, you safely dispose of the needle in a sharps disposal container (once disposal per week). - You consistently follow this guidance, and your weight is maintained at a level between being obese and overweight, without further increasing or decreasing. - Despite following the guidance, at this level, your risk of obesity-related complications is not significantly increased compared to a person who is overweight, but not obese, and you are more easily able to complete activities such as work. |
| --- |

**#2 GLP-1 RA QD (once-daily + oral therapy)**

| - You have obesity. - Because of your obesity, your doctor has advised you to monitor your diet and exercise regularly. - You follow your doctor’s recommendations about your diet. - You count the number of calories in the foods that you eat, and you make sure that you do not exceed the daily limit that your doctor has recommended. - For certain foods, you limit the amount you eat, or how often you eat them. - You follow your doctor’s recommendations about exercise. - You do aerobic exercise (such as brisk walking) for 2½ hours per week, spread across three or more days. - Your doctor has also advised you to take medication that reduces your weight. - You take tablets and injections. - You take daily tablets prescribed by your doctor. - You give yourself an injection **once a day**, from a pre-filled injection device. (Seven injections per week.) - You may choose the time of day when you give yourself the injection; however, this time of day should be kept consistent (for example, after dinner each evening). - Before beginning to use the injection device, you may store it in your refrigerator or at room temperature. - Before giving yourself the injection, you must place a new needle on the device and remove both protective caps from the needle. Because of the protective cap, you do not directly handle the needle at any point. You must then dial up to priming dose, and press the button to test whether the medicine can clearly pass through this needle. When this is confirmed, you can dial up to the dose that your doctor has advised, and then administer the injection. - After you give yourself the injection, you safely dispose of the needle in a sharps disposal container (one disposal per day). - You consistently follow this guidance, and your weight is maintained at a level between being obese and overweight, without further increasing or decreasing. - Despite following the guidance, at this level, your risk of obesity-related complications is not significantly increased compared to a person who is overweight, but not obese, and you are more easily able to complete activities such as work. |
| --- |

## T2DM introductory text and health state descriptions

**Introductory text**

Type 2 diabetes is a condition where the body does not produce enough insulin, or does not react properly to the insulin it does produce. (Insulin is the hormone that reduces the amount of sugar in a person’s blood.) Therefore, a person with this condition has uncontrolled levels of sugar in their blood compared to a person without diabetes. Uncontrolled levels of sugar in the blood increase the risk of complications that affect the heart, eyes, nerves, and other parts of the body. Therefore, reducing the level of sugar in the blood is positive for health.

**Anchor (diet and exercise only)**

| - You have type 2 diabetes. - Because of your diabetes, your doctor has advised you to monitor your diet and exercise regularly. - You follow your doctor’s recommendations about your diet. - You eat meals and snacks at regular times each day. - You also count the amount of carbohydrate in the foods that you eat, and match the amount of carbohydrate with your activity level each day. - For certain foods, you limit the amount you eat, or how often you eat them. - You follow your doctor’s recommendations about exercise. - You do aerobic exercise (such as brisk walking) for 2½ hours per week, spread across three or more days. - You also do 2-3 sessions of resistance or strength exercise per week (for example lifting a weight that is suitable for your level of fitness). - You consistently follow this guidance, and your blood sugar level is maintained at a controlled level similar to a person without diabetes, without further increasing or decreasing. - Despite following the guidance, at this level, you may experience temporary episodes where your blood sugar is too high or low. If your blood sugar is too high, you may feel tired and thirsty, and your vision may become blurred. If your blood sugar is too low, you may feel dizzy and shaky, and you may sweat excessively. You might require a food/drink source that contains high sugar, with more severe cases requiring hospitalisation. However, your risk of diabetes-related complications is not significantly increased compared to a person without diabetes. |
| --- |

**#1 GLP-1 RA QW (once-weekly + oral therapy)**

| - You have type 2 diabetes. - Because of your diabetes, your doctor has advised you to monitor your diet and exercise regularly. - You follow your doctor’s recommendations about your diet. - You eat meals and snacks at regular times each day. - You also count the amount of carbohydrate in the foods that you eat, and match the amount of carbohydrate with your activity level each day. - For certain foods, you limit the amount you eat, or how often you eat them. - You follow your doctor’s recommendations about exercise. - Your doctor has also advised you to take medication that reduces your blood sugar level. - You take a tablet and an injection. - You take one tablet a day with your evening meal. - You give yourself an injection **once a week**, from a pre-filled injection device. (One injection per week.) - You may choose the time of day when you give yourself the injection. - Before beginning to use the injection device, you may store it in your refrigerator or at room temperature. - Before giving yourself the injection, you must place a new needle on the device and remove both protective caps from the needle. Because of the protective cap, you do not directly handle the needle at any point. You must then dial up to priming dose, and press the button to test whether the medicine can clearly pass through this needle. When this is confirmed, you can dial up to the dose that your doctor has advised, and then administer the injection. - After you give yourself the injection, you safely dispose of the needle in a sharps disposal container (once disposal per week). - You do aerobic exercise (such as brisk walking) for 2½ hours per week, spread across three or more days. - You also do 2-3 sessions of resistance or strength exercise per week (for example lifting a weight that is suitable for your level of fitness). - You consistently follow this guidance, and your blood sugar level is maintained at a controlled level similar to a person without diabetes, without further increasing or decreasing. - Despite following the guidance, at this level, you may experience temporary episodes where your blood sugar is too high or low. If your blood sugar is too high, you may feel tired and thirsty, and your vision may become blurred. If your blood sugar is too low, you may feel dizzy and shaky, and you may sweat excessively. You might require a food/drink source that contains high sugar, with more severe cases requiring hospitalisation. However, your risk of diabetes-related complications is not significantly increased compared to a person without diabetes. |
| --- |

**#2 GLP-1 RA QD (once-daily + oral therapy)**

| - You have type 2 diabetes. - Because of your diabetes, your doctor has advised you to monitor your diet and exercise regularly. - You follow your doctor’s recommendations about your diet. - You eat meals and snacks at regular times each day. - You also count the amount of carbohydrate in the foods that you eat, and match the amount of carbohydrate with your activity level each day. - For certain foods, you limit the amount you eat, or how often you eat them. - You follow your doctor’s recommendations about exercise. - Your doctor has also advised you to take medication that reduces your blood sugar level. - You take tablets and injections. - You take one tablet a day with your evening meal. - You give yourself an injection **once a day**, from a pre-filled injection device. (Seven injections per week.) - You may choose the time of day when you give yourself the injection; however, this time of day should be kept consistent (for example, after dinner each evening). - Before beginning to use the injection device, you may store it in your refrigerator or at room temperature. - Before giving yourself the injection, you must place a new needle on the device and remove both protective caps from the needle. Because of the protective cap, you do not directly handle the needle at any point. You must then dial up to priming dose, and press the button to test whether the medicine can clearly pass through this needle. When this is confirmed, you can dial up to the dose that your doctor has advised, and then administer the injection. - After you give yourself the injection, you safely dispose of the needle in a sharps disposal container (one disposal per day). - You do aerobic exercise (such as brisk walking) for 2½ hours per week, spread across three or more days. - You also do 2-3 sessions of resistance or strength exercise per week (for example lifting a weight that is suitable for your level of fitness). - You consistently follow this guidance, and your blood sugar level is maintained at a controlled level similar to a person without diabetes, without further increasing or decreasing. - Despite following the guidance, at this level, you may experience temporary episodes where your blood sugar is too high or low. If your blood sugar is too high, you may feel tired and thirsty, and your vision may become blurred. If your blood sugar is too low, you may feel dizzy and shaky, and you may sweat excessively. You might require a food/drink source that contains high sugar, with more severe cases requiring hospitalisation. However, your risk of diabetes-related complications is not significantly increased compared to a person without diabetes. |
| --- |

**#3 Insulin QW (once-weekly, with once-weekly blood test + oral therapy)**

| - You have type 2 diabetes. - Because of your diabetes, your doctor has advised you to monitor your diet and exercise regularly. - You follow your doctor’s recommendations about your diet. - You eat meals and snacks at regular times each day. - You also count the amount of carbohydrate in the foods that you eat, and match the amount of carbohydrate with your activity level each day. - For certain foods, you limit the amount you eat, or how often you eat them. - You follow your doctor’s recommendations about exercise. - Your doctor has also advised you to take medication that reduces your blood sugar level. - You take tablets and injections. - You take one tablet a day with your evening meal. - You check your blood sugar level once a week using a portable blood glucose meter, called a glucometer. This process uses a finger-prick test. Using a lancet, it lightly pricks your skin to obtain a small amount of blood. The meter tells you your current blood sugar. - You give yourself an injection **once a week**, from a pre-filled injection device. (One injection per week.) - You may choose the time of day when you give yourself the injection. - Before beginning to use the injection device, you may store it in your refrigerator or at room temperature. - Before giving yourself the injection, you must place a new needle on the device and remove both protective caps from the needle. Because of the protective cap, you do not directly handle the needle at any point. You must then dial up to priming dose, and press the button to test whether the medicine can clearly pass through this needle. When this is confirmed, you can dial up to the dose that your doctor has advised, and then administer the injection. - After you give yourself the injection, you safely dispose of the needle in a sharps disposal container (once disposal per week). - You do aerobic exercise (such as brisk walking) for 2½ hours per week, spread across three or more days. - You also do 2-3 sessions of resistance or strength exercise per week (for example lifting a weight that is suitable for your level of fitness). - You consistently follow this guidance, and your blood sugar level is maintained at a controlled level similar to a person without diabetes, without further increasing or decreasing. - Despite following the guidance, at this level, you may experience temporary episodes where your blood sugar is too high or low. If your blood sugar is too high, you may feel tired and thirsty, and your vision may become blurred. If your blood sugar is too low, you may feel dizzy and shaky, and you may sweat excessively. You might require a food/drink source that contains high sugar, with more severe cases requiring hospitalisation. However, your risk of diabetes-related complications is not significantly increased compared to a person without diabetes. |
| --- |

**#4 Insulin QD (once-daily, with once-daily blood test + oral therapy)**

| - You have type 2 diabetes. - Because of your diabetes, your doctor has advised you to monitor your diet and exercise regularly. - You follow your doctor’s recommendations about your diet. - You eat meals and snacks at regular times each day. - You also count the amount of carbohydrate in the foods that you eat, and match the amount of carbohydrate with your activity level each day. - For certain foods, you limit the amount you eat, or how often you eat them. - You follow your doctor’s recommendations about exercise. - Your doctor has also advised you to take medication that reduces your blood sugar level. - You take tablets and injections. - You take one tablet a day with your evening meal. - You check your blood sugar level once a day using a portable blood glucose meter, called a glucometer. This process uses a finger-prick test. Using a lancet, it lightly pricks your skin to obtain a small amount of blood. The meter tells you your current blood sugar. - You give yourself an injection **once a day**, from a pre-filled injection device. (Seven injections per week.) - You may choose the time of day when you give yourself the injection; however, this time of day should be kept consistent (for example, after dinner each evening). - Before beginning to use the injection device, you may store it in your refrigerator or at room temperature. - Before giving yourself the injection, you must place a new needle on the device and remove both protective caps from the needle. Because of the protective cap, you do not directly handle the needle at any point. You must then dial up to priming dose, and press the button to test whether the medicine can clearly pass through this needle. When this is confirmed, you can dial up to the dose that your doctor has advised, and then administer the injection. - After you give yourself the injection, you safely dispose of the needle in a sharps disposal container (one disposal per day). - You do aerobic exercise (such as brisk walking) for 2½ hours per week, spread across three or more days. - You also do 2-3 sessions of resistance or strength exercise per week (for example lifting a weight that is suitable for your level of fitness). - You consistently follow this guidance, and your blood sugar level is maintained at a controlled level similar to a person without diabetes, without further increasing or decreasing. - Despite following the guidance, at this level, you may experience temporary episodes where your blood sugar is too high or low. If your blood sugar is too high, you may feel tired and thirsty, and your vision may become blurred. If your blood sugar is too low, you may feel dizzy and shaky, and you may sweat excessively. You might require a food/drink source that contains high sugar, with more severe cases requiring hospitalisation. However, your risk of diabetes-related complications is not significantly increased compared to a person without diabetes. |
| --- |

**#4 Insulin BB (4× daily, with 4 × daily blood test + oral therapy)**

| - You have type 2 diabetes. - Because of your diabetes, your doctor has advised you to monitor your diet and exercise regularly. - You follow your doctor’s recommendations about your diet. - You eat meals and snacks at regular times each day. - You also count the amount of carbohydrate in the foods that you eat, and match the amount of carbohydrate with your activity level each day. - For certain foods, you limit the amount you eat, or how often you eat them. - You follow your doctor’s recommendations about exercise. - Your doctor has also advised you to take medication that reduces your blood sugar level. - You take tablets and injections. - You take one tablet a day with your evening meal. - You check your blood sugar level four times a day using a portable blood glucose meter, called a glucometer. This process uses a finger-prick test. Using a lancet, it lightly pricks your skin to obtain a small amount of blood. The meter tells you your current blood sugar. - You give yourself an injection **four times a day**, from pre-filled injection devices. (Twenty-eight injections per week.) - You take an injection from one device before each main meal (breakfast, lunch, and dinner). You also take another injection from another device at a time of day that you choose; however, this time of day should be kept consistent (for example, after dinner each evening). - Before beginning to use the injection device, you may store it in your refrigerator or at room temperature. - Before giving yourself the injection, you must place a new needle on the device and remove both protective caps from the needle. Because of the protective cap, you do not directly handle the needle at any point. You must then dial up to priming dose, and press the button to test whether the medicine can clearly pass through this needle. When this is confirmed, you can dial up to the dose that your doctor has advised, and then administer the injection. - After you give yourself the injection, you safely dispose of the needle in a sharps disposal container (four times a day). - You do aerobic exercise (such as brisk walking) for 2½ hours per week, spread across three or more days. - You also do 2-3 sessions of resistance or strength exercise per week (for example lifting a weight that is suitable for your level of fitness). - You consistently follow this guidance, and your blood sugar level is maintained at a controlled level similar to a person without diabetes, without further increasing or decreasing. - Despite following the guidance, at this level, you may experience temporary episodes where your blood sugar is too high or low. If your blood sugar is too high, you may feel tired and thirsty, and your vision may become blurred. If your blood sugar is too low, you may feel dizzy and shaky, and you may sweat excessively. You might require a food/drink source that contains high sugar, with more severe cases requiring hospitalisation. However, your risk of diabetes-related complications is not significantly increased compared to a person without diabetes. |
| --- |

## Further detailed TTO utility results

Table 7. TTO utility values in obesity and T2DM, in the UK, Canada, and China

| UK (n=110) | | | | | | | | | |
| --- | --- | --- | --- | --- | --- | --- | --- | --- | --- |
|  | **Obesity** | | | **T2DM** | | | | | |
|  | **Anchor** | **GLP-1 RA** | | **Anchor** | **GLP-1 RA** | | **Insulin** | | |
|  |  | **QW** | **QD** |  | **QW** | **QD** | **QW** | **QD** | **BB** |
| **Mean** | 0.877 | 0.857 | 0.816 | 0.888 | 0.877 | 0.845 | 0.866 | 0.827 | 0.741 |
| **SD** | 0.165 | 0.176 | 0.194 | 0.161 | 0.177 | 0.180 | 0.179 | 0.183 | 0.210 |
| **Median** | 0.950 | 0.900 | 0.898 | 0.950 | 0.950 | 0.900 | 0.904 | 0.869 | 0.800 |
| **IQR** | 0.840 | 0.825 | 0.750 | 0.875 | 0.877 | 0.800 | 0.825 | 0.800 | 0.693 |
|  | 0.996 | 0.950 | 0.950 | 0.996 | 0.963 | 0.950 | 0.970 | 0.950 | 0.900 |
| **Canada (n=100)** | | | | | | | | | |
|  | **Obesity** | | | **T2DM** | | | | | |
|  | **Anchor** | **GLP-1 RA** | | **Anchor** | **GLP-1 RA** | | **Insulin** | | |
|  |  | **QW** | **QD** |  | **QW** | **QD** | **QW** | **QD** | **BB** |
| **Mean** | 0.843 | 0.790 | 0.757 | 0.872 | 0.842 | 0.805 | 0.822 | 0.765 | 0.669 |
| **SD** | 0.222 | 0.253 | 0.268 | 0.200 | 0.216 | 0.233 | 0.229 | 0.263 | 0.297 |
| **Median** | 0.913 | 0.900 | 0.800 | 0.950 | 0.900 | 0.900 | 0.900 | 0.838 | 0.700 |
| **IQR** | 0.800 | 0.700 | 0.700 | 0.800 | 0.788 | 0.700 | 0.744 | 0.688 | 0.500 |
|  | 1.000 | 0.993 | 0.951 | 1.000 | 1.000 | 1.000 | 1.000 | 0.996 | 0.900 |
| **China (n=100)** | | | | | | | | | |
|  | **Obesity** | | | **T2DM** | | | | | |
|  | **Anchor** | **GLP-1 RA** | | **Anchor** | **GLP-1 RA** | | **Insulin** | | |
|  |  | **QW** | **QD** |  | **QW** | **QD** | **QW** | **QD** | **BB** |
| **Mean** | 0.939 | 0.869 | 0.774 | 0.921 | 0.863 | 0.773 | 0.855 | 0.760 | 0.637 |
| **SD** | 0.091 | 0.146 | 0.198 | 0.103 | 0.146 | 0.200 | 0.153 | 0.204 | 0.249 |
| **Median** | 0.975 | 0.900 | 0.800 | 0.950 | 0.900 | 0.800 | 0.900 | 0.800 | 0.700 |
| **IQR** | 0.900 | 0.800 | 0.700 | 0.900 | 0.800 | 0.700 | 0.800 | 0.700 | 0.500 |
|  | 0.996 | 0.966 | 0.900 | 0.996 | 0.950 | 0.922 | 0.950 | 0.900 | 0.813 |

BB: basal-bolus; GLP-1 RA: glucagon-like peptide-1 receptor agonist; IQR: interquartile range; QD: daily; QW: weekly; SD: standard deviation; TTO: time trade-off.

Figure 2: TTO utility values in obesity and T2DM in the UK (n=110)


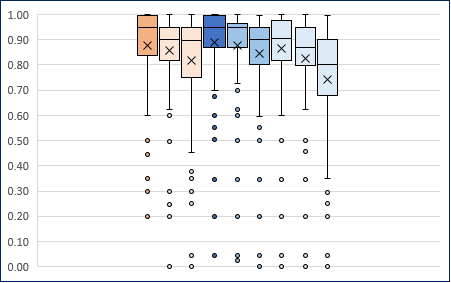


Order of presentation: obesity anchor state, GLP-1 RA QW, GLP-1 RA QD; T2DM anchor state, GLP-1 RA QW, GLP-1 RA QD, insulin QW, insulin QD, insulin BB; × mean, — median value, ◻ interquartile range (first quartile to third quartile),

○ outlier value (outside 1.5× interquartile range).

Figure 3: TTO utility values in obesity and T2DM in Canada (n=100)


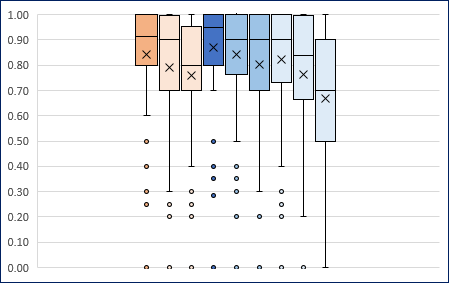


Order of presentation: obesity anchor state, GLP-1 RA QW, GLP-1 RA QD; T2DM anchor state, GLP-1 RA QW, GLP-1 RA QD, insulin QW, insulin QD, insulin BB; × mean, — median value, ◻ interquartile range (first quartile to third quartile),

○ outlier value (outside 1.5× interquartile range).

Figure 4: TTO utility values in obesity and T2DM in China (n=100)


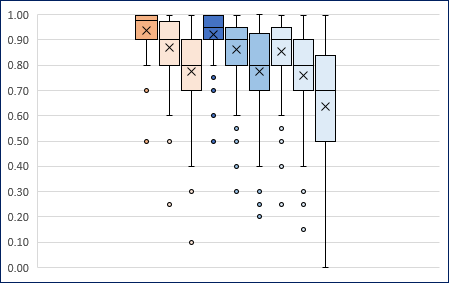


Order of presentation: obesity anchor state, GLP-1 RA QW, GLP-1 RA QD; T2DM anchor state, GLP-1 RA QW, GLP-1 RA QD, insulin QW, insulin QD, insulin BB; × mean, — median value, ◻ interquartile range (first quartile to third quartile),

○ outlier value (outside 1.5× interquartile range).

## Subgroup analyses of TTO utility results

Table 8. Utility values elicited by TTO from participant subgroups in the UK

|  | Obesity | | | T2DM | | | | | |
| --- | --- | --- | --- | --- | --- | --- | --- | --- | --- |
|  | **Anchor** | **GLP-1 RA** | | **Anchor** | **GLP-1 RA** | | **Insulin** | | |
|  |  | **QW** | **QD** |  | **QW** | **QD** | **QW** | **QD** | **BB** |
| **Full sample *(base case)***  (n=110); mean | 0.877 | 0.857 | 0.816 | 0.888 | 0.877 | 0.845 | 0.866 | 0.827 | 0.741 |
| **No 0.0 or 1.0***  (n=*); mean | 0.828 | 0.840 | 0.798 | 0.840 | 0.847 | 0.825 | 0.837 | 0.807 | 0.726 |
| **No high/low 5%**  (n=98†; mean) | 0.902 | 0.883 | 0.842 | 0.914 | 0.907 | 0.871 | 0.895 | 0.852 | 0.763 |
| **No +/−2 SD**  (n=‡; mean) | 0.911 | 0.893 | 0.851 | 0.918 | 0.912 | 0.871 | 0.901 | 0.857 | 0.776 |
| **No illogical**  (n=§; mean) | 0.877 | 0.863 | 0.817 | 0.888 | 0.872 | 0.845 | 0.862 | 0.825 | 0.739 |
| **Male**  (n=51); mean | 0.850 | 0.831 | 0.784 | 0.883 | 0.874 | 0.844 | 0.866 | 0.824 | 0.711 |
| **Female**  (n=59); mean | 0.900 | 0.879 | 0.844 | 0.893 | 0.879 | 0.846 | 0.831 | 0.767 | 0.893 |
| **Age <40 years**  (n=48); mean | 0.886 | 0.864 | 0.825 | 0.895 | 0.885 | 0.840 | 0.872 | 0.825 | 0.722 |
| **Age ≥40 years**  (n=62); mean | 0.870 | 0.851 | 0.810 | 0.883 | 0.870 | 0.849 | 0.862 | 0.829 | 0.756 |

* “*No 0.0 or 1.0*” analysis does not include any utility value of 0.0 (equivalent to death) or 1.0 (equivalent to full health); 177 of 990 total health state valuations were removed for this analysis – therefore, n differs between health states.

† *“No high/low 5%”* analysis does not include the highest 5% or lowest 5% of utility values for each health state; therefore, the total number of valuations per health state is 98.

‡ *“No +/−2 SD”* analysis does not include any utility value that is two or more standard deviations above or below the corresponding health state mean; therefore, n differs between health states.

§ *“No illogical”* analysis does not include any utility value that was deemed to be illogical as per a logic check (see Methods); therefore, n differs between health states.

Table 9. Utility values elicited by TTO from participant subgroups in Canada

|  | Obesity | | | T2DM | | | | | |
| --- | --- | --- | --- | --- | --- | --- | --- | --- | --- |
|  | **Anchor** | **GLP-1 RA** | | **Anchor** | **GLP-1 RA** | | **Insulin** | | |
|  |  | **QW** | **QD** |  | **QW** | **QD** | **QW** | **QD** | **BB** |
| **Full sample *(base case)***  (n=100); mean | 0.843 | 0.790 | 0.757 | 0.872 | 0.842 | 0.805 | 0.822 | 0.765 | 0.669 |
| **No 0.0 or 1.0***  (n=*); mean | 0.768 | 0.761 | 0.747 | 0.836 | 0.792 | 0.753 | 0.774 | 0.733 | 0.678 |
| **No high/low 5%**  (n=90†; mean) | 0.872 | 0.820 | 0.786 | 0.901 | 0.869 | 0.831 | 0.850 | 0.792 | 0.688 |
| **No +/−2 SD**  (n=‡; mean) | 0.894 | 0.843 | 0.812 | 0.907 | 0.888 | 0.839 | 0.864 | 0.816 | 0.727 |
| **No illogical**  (n=§; mean) | 0.843 | 0.788 | 0.757 | 0.872 | 0.831 | 0.809 | 0.819 | 0.764 | 0.666 |
| **Male**  (n=49); mean | 0.807 | 0.769 | 0.724 | 0.874 | 0.842 | 0.795 | 0.813 | 0.737 | 0.643 |
| **Female**  (n=51); mean | 0.876 | 0.811 | 0.790 | 0.869 | 0.842 | 0.814 | 0.830 | 0.792 | 0.694 |
| **Age <40 years**  (n=36); mean | 0.783 | 0.736 | 0.698 | 0.837 | 0.796 | 0.748 | 0.770 | 0.724 | 0.631 |
| **Age ≥40 years**  (n=64); mean | 0.879 | 0.824 | 0.794 | 0.893 | 0.870 | 0.839 | 0.853 | 0.790 | 0.693 |

* “*No 0.0 or 1.0*” analysis does not include any utility value of 0.0 (equivalent to death) or 1.0 (equivalent to full health); 298 of 900 total health state valuations were removed for this analysis – therefore, n differs between health states.

† *“No high/low 5%”* analysis does not include the highest 5% or lowest 5% of utility values for each health state; therefore, the total number of valuations per health state is 90.

‡ *“No +/−2 SD”* analysis does not include any utility value that is two or more standard deviations above or below the corresponding health state mean; therefore, n differs between health states.

§ *“No illogical”* analysis does not include any utility value that was deemed to be illogical as per a logic check (see Methods); therefore, n differs between health states.

Table 10. Utility values elicited by TTO from participant subgroups in China

|  | Obesity | | | T2DM | | | | | |
| --- | --- | --- | --- | --- | --- | --- | --- | --- | --- |
|  | **Anchor** | **GLP-1 RA** | | **Anchor** | **GLP-1 RA** | | **Insulin** | | |
|  |  | **QW** | **QD** |  | **QW** | **QD** | **QW** | **QD** | **BB** |
| **Full sample *(base case)***  (n=100); mean | 0.939 | 0.869 | 0.774 | 0.921 | 0.863 | 0.773 | 0.855 | 0.760 | 0.637 |
| **No 0.0 or 1.0***  (n=*); mean | 0.903 | 0.835 | 0.741 | 0.888 | 0.828 | 0.737 | 0.818 | 0.725 | 0.627 |
| **No high/low 5%**  (n=90†; mean) | 0.953 | 0.888 | 0.790 | 0.936 | 0.879 | 0.790 | 0.872 | 0.778 | 0.650 |
| **No +/−2 SD**  (n=‡; mean) | 0.955 | 0.893 | 0.792 | 0.939 | 0.893 | 0.806 | 0.890 | 0.795 | 0.663 |
| **No illogical**  (n=§; mean) | 0.939 | 0.869 | 0.774 | 0.921 | 0.863 | 0.771 | 0.855 | 0.759 | 0.637 |
| **Male**  (n=50); mean | 0.958 | 0.894 | 0.790 | 0.916 | 0.860 | 0.753 | 0.852 | 0.741 | 0.619 |
| **Female**  (n=50); mean | 0.919 | 0.843 | 0.757 | 0.926 | 0.867 | 0.793 | 0.858 | 0.780 | 0.656 |
| **Age <40 years**  (n=70); mean | 0.936 | 0.857 | 0.767 | 0.918 | 0.855 | 0.760 | 0.847 | 0.746 | 0.626 |
| **Age ≥40 years**  (n=30); mean | 0.944 | 0.892 | 0.787 | 0.930 | 0.882 | 0.804 | 0.875 | 0.794 | 0.665 |

* “*No 0.0 or 1.0*” analysis does not include any utility value of 0.0 (equivalent to death) or 1.0 (equivalent to full health); 183 of 900 total health state valuations were removed for this analysis – therefore, n differs between health states.

† “No high/low 5%” analysis does not include the highest 5% or lowest 5% of utility values for each health state; therefore, the total number of valuations per health state is 90.

‡ *“No +/−2 SD”* analysis does not include any utility value that is two or more standard deviations above or below the corresponding health state mean; therefore, n differs between health states.

§ *“No illogical”* analysis does not include any utility value that was deemed to be illogical as per a logic check (see Methods); therefore, n differs between health states.

## Detailed VAS scores

Figure 5: Scores by VAS (converted to utility values) from participants in the UK (n=110)


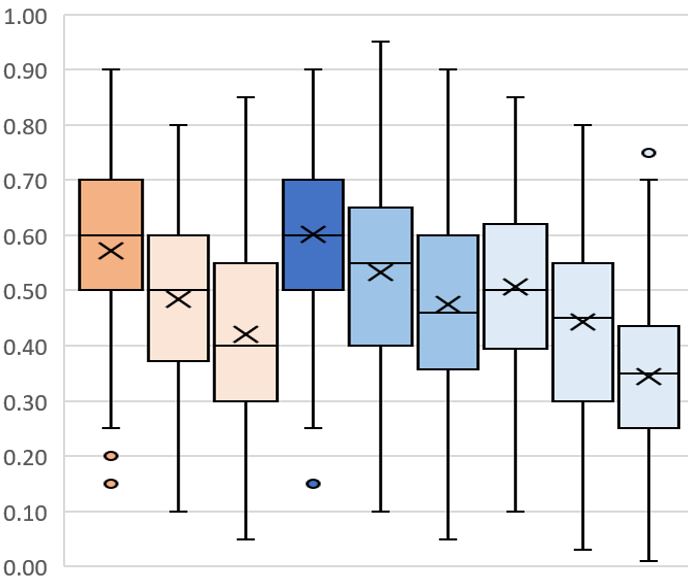


Tested health states were obesity anchor state, GLP-1 RA QW, GLP-1 RA QD;

T2DM anchor state, GLP-1 RA QW, GLP-1 RA QD, insulin QW, insulin QD, insulin BB;

× mean, — median value, ◻ interquartile range (first quartile to third quartile),

○ outlier value (outside 1.5× interquartile range).

Figure 6. Scores by VAS (converted to utility values) from participants in Canada (n=100)


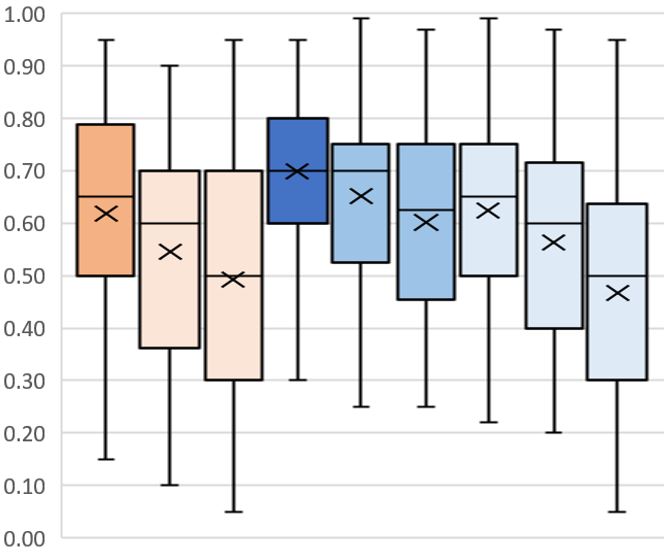


Tested health states were obesity anchor state, GLP-1 RA QW, GLP-1 RA QD;

T2DM anchor state, GLP-1 RA QW, GLP-1 RA QD, insulin QW, insulin QD, insulin BB;

× mean, — median value, ◻ interquartile range (first quartile to third quartile),

○ outlier value (outside 1.5× interquartile range).

Figure 7: Scores by VAS (converted to utility values) from participants in China (n=100)


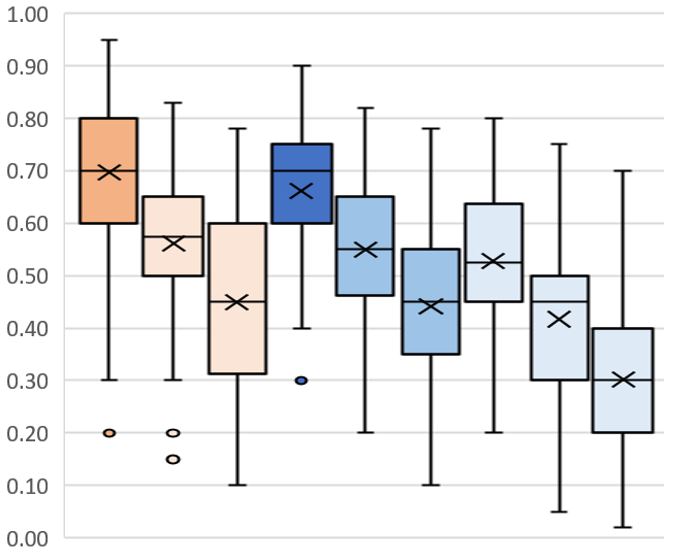


Tested health states were obesity anchor state, GLP-1 RA QW, GLP-1 RA QD;

T2DM anchor state, GLP-1 RA QW, GLP-1 RA QD, insulin QW, insulin QD, insulin BB;

× mean, — median value, ◻ interquartile range (first quartile to third quartile),

○ outlier value (outside 1.5× interquartile range).

Table 11. VAS scores elicited from participants in the UK (n=110)

|  | Obesity | | | T2DM | | | | | |
| --- | --- | --- | --- | --- | --- | --- | --- | --- | --- |
|  | **Anchor** | **GLP-1 RA** | | **Anchor** | **GLP-1 RA** | | **Insulin** | | |
|  |  | **QW** | **QD** |  | **QW** | **QD** | **QW** | **QD** | **BB** |
| **Mean** | 57.22 | 48.37 | 42.05 | 60.24 | 53.35 | 47.42 | 50.57 | 44.23 | 34.38 |
| **SD** | 16.84 | 16.73 | 18.15 | 15.73 | 16.22 | 16.88 | 16.66 | 16.59 | 15.87 |
| **Median** | 60.00 | 50.00 | 40.00 | 60.00 | 55.00 | 46.00 | 50.00 | 45.00 | 35.00 |
| **IQR** | 50.00 | 38.00 | 30.00 | 50.00 | 40.00 | 36.25 | 40.00 | 31.25 | 25.00 |
|  | 70.00 | 60.00 | 55.00 | 70.00 | 65.00 | 60.00 | 61.50 | 55.00 | 42.25 |

BB: basal-bolus; GLP-1 RA: glucagon-like peptide-1 receptor agonist; IQR: interquartile range; QD: daily; QW: weekly; SD: standard deviation; VAS: visual analogue scale.

Table 12. VAS scores elicited from participants in Canada (n=100)

|  | Obesity | | | T2DM | | | | | |
| --- | --- | --- | --- | --- | --- | --- | --- | --- | --- |
|  | **Anchor** | **GLP-1 RA** | | **Anchor** | **GLP-1 RA** | | **Insulin** | | |
|  |  | **QW** | **QD** |  | **QW** | **QD** | **QW** | **QD** | **BB** |
| **Mean** | 61.86 | 54.56 | 49.30 | 69.88 | 65.15 | 60.14 | 62.45 | 56.33 | 46.69 |
| **SD** | 18.46 | 18.91 | 19.51 | 15.65 | 16.31 | 18.15 | 17.11 | 19.31 | 21.06 |
| **Median** | 65.00 | 60.00 | 50.00 | 70.00 | 70.00 | 62.50 | 65.00 | 60.00 | 50.00 |
| **IQR** | 50.00 | 38.75 | 30.00 | 60.00 | 53.50 | 46.50 | 50.00 | 40.00 | 30.00 |
|  | 76.25 | 70.00 | 70.00 | 80.00 | 75.00 | 75.00 | 75.00 | 70.50 | 61.25 |

BB: basal-bolus; GLP-1 RA: glucagon-like peptide-1 receptor agonist; IQR: interquartile range; QD: daily; QW: weekly; SD: standard deviation; VAS: visual analogue scale.

Table 13. VAS scores elicited from participants in China (n=100)

|  | Obesity | | | T2DM | | | | | |
| --- | --- | --- | --- | --- | --- | --- | --- | --- | --- |
|  | **Anchor** | **GLP-1 RA** | | **Anchor** | **GLP-1 RA** | | **Insulin** | | |
|  |  | **QW** | **QD** |  | **QW** | **QD** | **QW** | **QD** | **BB** |
| **Mean** | 69.67 | 56.06 | 44.97 | 66.15 | 54.99 | 44.16 | 52.82 | 41.70 | 30.15 |
| **SD** | 14.22 | 14.50 | 15.90 | 12.62 | 13.36 | 14.73 | 13.42 | 15.38 | 14.63 |
| **Median** | 70.00 | 57.50 | 45.00 | 70.00 | 55.00 | 45.00 | 52.50 | 45.00 | 30.00 |
| **IQR** | 60.00 | 50.00 | 33.75 | 60.00 | 46.75 | 35.00 | 45.00 | 30.00 | 20.00 |
|  | 80.00 | 65.00 | 60.00 | 75.00 | 65.00 | 55.00 | 61.25 | 50.00 | 40.00 |

BB: basal-bolus; GLP-1 RA: glucagon-like peptide-1 receptor agonist; IQR: interquartile range; QD: daily; QW: weekly; SD: standard deviation; VAS: visual analogue scale.
